# Long Chain Polyunsaturated Fatty Acids Alter Oxytocin Signaling and Receptor Density in Cultured Pregnant Human Myometrial Smooth Muscle Cells

**DOI:** 10.1371/journal.pone.0041708

**Published:** 2012-07-26

**Authors:** Paul Y. Kim, Miao Zhong, Yoon-Sun Kim, Barbara M. Sanborn, Kenneth G. D. Allen

**Affiliations:** 1 Department of Food Science and Human Nutrition, Colorado State University, Fort Collins, Colorado, United States of America; 2 Department of Biomedical Sciences, Colorado State University, Fort Collins, Colorado, United States of America; Fudan University, China

## Abstract

Epidemiological studies and interventional clinical trials indicate that consumption of long chain n-3 polyunsaturated fatty acids (LC n-3 PUFA) such as docosahexaenoic acid (DHA) lengthen gestational duration. Although the mechanisms are not well understood, prostaglandins (PG) of the 2-series are known to play a role in the initiation and progress of labor. In animal studies, modest DHA provision has been shown to reduce placental and uterine PGE_2_ and PGF_2α_, matrix metalloproteinase (MMP)-2 and MMP-9 expression, and placental collagenase activity. However, modulation of PG biosynthesis may not account for all the effects of LC n-3 PUFAs in labor. We investigated one potential PG-independent mechanism of LC PUFA action using cultured pregnant human myometrial smooth muscle cells. Our goal was to characterize the effect of LC PUFA treatment on oxytocin signaling, a potent uterotonic hormone involved in labor. The addition of 10 µM–100 µM DHA or arachidonic acid (AA) to the culture media for 48 h resulted in dose dependent enrichment of these fatty acids in membrane lipid. DHA and AA significantly inhibited phosphatidylinositol turnover and [Ca^2+^]_i_ mobilization with oxytocin stimulation compared to bovine serum albumin control and equimolar oleic acid. DHA and AA significantly reduced oxytocin receptor membrane concentration without altering binding affinity or rate of receptor internalization. These findings demonstrate a role for LC n-3 PUFAs in regulation of oxytocin signaling and provide new insight into additional mechanisms pertaining to reports of dietary fish and fish oil consumption prolonging gestation.

## Introduction

The Centers for Disease Control reports that the percentage of infants delivered preterm (<37 weeks of completed gestation) increased sharply from 9.4% in 1981 to 12% in 2010. The percentage of infants born low birth weight (<2500 g) rose from 6.6% to 8.15% during this period [1]. Analysis of gestational duration from 1992 to 2002 has shown a shift in median duration from 40 weeks in 1992 to 39 weeks in 2002, which was accompanied by marked significant decreases in deliveries at ≥40 weeks and increases in those at 34 to 39 weeks [2]. Shortened gestation and low birth weight are major risk factors for death and morbidity in infants [2]–[4]. While medical advances have improved preterm infant outcomes, efforts to prevent preterm birth have been much less successful.

Evidence from observational and clinical studies indicate that essential fatty acids of the n-3 and n-6 series may potentially modify gestational duration [5]. Early epidemiological studies in Denmark by Olsen and colleagues found a significant increase in gestational duration with increasing maternal erythrocyte long-chain n-3 polyunsaturated fatty acids (LC n-3 PUFA) [6], [7]. In subsequent randomized control trials, LC n-3 PUFA supplementation during pregnancy has been shown to increase gestational duration by 4 to 6 days [8]–[12]. These findings have recently been replicated by others [13], [14] and are supported by a recent systematic review and meta-analysis [15]. The mechanism remains largely unknown.

Evidence indicates that prostaglandins (PGs) play a key role in initiating and maintaining normal labor [16]. PGE_2_ and PGF_2α_ increase at term in laboring women compared with nonlaboring women [17], [18], can activate tissue remodeling matrix metalloproteinases [19]–[21], mediate cervical ripening [22], [23], and induce uterine contractions [24], [25]. These PG_2_-mediated actions coupled with markedly elevated arachidonic acid (AA) in maternal erythrocytes of women delivering prematurely [26], apparent LC n-3 PUFA deficiency in these women [26], and the opposing effects of LC n-3 PUFAs and AA on PG_2_ biosynthesis [27]–[29] have pointed investigators toward a mechanism in which LC n-3 PUFAs prolong pregnancy by suppressing the AA cascade [8], [9], [12]. Elevated reproductive tissue cytokine and PG production have been implicated in preterm birth involving intrauterine infection [30], but the role of PGs in idiopathic preterm labor is less well defined. In the absence of infection, PG concentrations in chorion, decidua, and myometrium are reported to be lower in women delivering prematurely compared with women delivering at term [31]–[33]. Although the threshold for effectiveness has not been established, these findings suggest that mechanisms other than or in addition to PG suppression may account for the observed increase in gestational duration with LC n-3 PUFA supplementation in expectant women [12].

Independent of PG biosynthesis, LC n-3 PUFAs have been shown to exert their cardioprotective [34] and immunosuppressive [35], [36] effects at the level of the plasma membrane. Docosahexanoic acid (DHA)-induced changes in the membrane structure and architecture leading to modulation of ion channels and the displacement of key proteins from lipid raft signaling platforms appear to be the mechanisms of action [37]–[39]. Manipulation of the lipid environment has also been shown to modulate the action of oxytocin [40]–[45], a potent uterotonic hormone involved in labor [46]. Binding of oxytocin to its plasma membrane receptor activates phospholipase C to produce inositol 1,4,5-trisphosphate (IP_3_) from the hydrolysis of phosphatidylinositol bisphosphate. Activation of the IP_3_ receptor releases Ca^2+^ from intracellular stores, resulting in an increase in the concentration of the calcium-calmodulin complex. In smooth muscle, this complex activates myosin light chain kinase, which phosphorylates the 20 kDa light chain of myosin and facilitates contraction. In humans, myometrial sensitivity to oxytocin is significantly increased in women delivering preterm and decreased in women delivering post-term [47].

To complement the observations of lengthened gestation with DHA and fish oil supplementation and demonstrate one potential PG-independent mechanism, we examined the effect of LC PUFA on oxytocin signaling in pregnant human myometrial cells. The goal of our study was to characterize changes in the cell membrane lipid composition in response to the addition of micromolar concentrations of the free fatty acids oleic (OA), AA, and DHA into the culture media and to determine whether incorporation of these exogenous fatty acids into the myometrial plasma membrane affects oxytocin-mediated signaling events involved in smooth muscle contraction.

## Materials and Methods

### Reagents

Dulbecco's Modified Eagle's Medium (DMEM), fetal bovine serum (FBS), trypsin, and other cell culture reagents were acquired from HyClone (Logan, UT). Fatty acid-free bovine serum albumin (BSA) was obtained from Sigma-Aldrich (St. Louis, MO). Fatty acids and fatty acid methyl ester standards were from Nu-Chek Prep (Elysian, MN). Oxytocin and other compounds were from Sigma-Aldrich. [^3^H]-myoinositol and [^3^H]-oxytocin were from Perkin Elmer New England Nuclear (Waltham, MA).

### Cell culture

Immortalized pregnant human myometrial cells (PHM1-41) retain many morphological and phenotypic responses of primary myometrial cells, express smooth muscle-specific α-actin, and retain oxytocin receptors [48]–[52]. PHM1-41 cells were maintained in DMEM supplemented with 10% FBS and 2 mM L-glutamine in a humidified atmosphere of 5% CO_2_ at 37°C. Cells used in the study were between passages 20–25.

### Fatty acid supplementation

Stock solutions of the fatty acids 18∶1 oleic acid (OA), 20∶4 arachidonic acid (AA), and 22∶6 docosahexanoic acid (DHA) were prepared in absolute ethanol at a concentration of 0.5 M, purged in argon gas, and stored at −20°C. 100 µM fatty acid media was prepared by adding fatty acid free BSA to cell culture media to a final BSA concentration of 50 µM, then incubating overnight with 2 µl of 0.5 M fatty acid stock solution per 10 ml media. 30 µM and 10 µM fatty acid media were prepared by dilution of 100 µM fatty acid media with BSA-free media. BSA-only media served as our control. The final ethanol concentration was adjusted to 0.02%. Fatty acid composition in the final media was confirmed by gas chromatography (see below). For all experiments, the cells were placed in fatty acid or control media 48 h after subculture; cells were grown for an additional 48 h and the treatment media were completely removed prior to assays.

### Cell membrane preparation

PHM1-41 cells were seeded at a density of 5.5×10^5^ cells/dish in 100 mm tissue culture dishes for 4 days. After rinsing twice with cold phosphate buffered saline (PBS), cells were harvested in ice-cold hypotonic buffer (20 mM HEPES, 10 mM EDTA, pH 7.4) as described previously [53]. Cells were mechanically disrupted by 4×15 sec 25,000-rpm pulses with an Ultra Turrax T8 homogenizer at 4°C. Cell homogenates were centrifuged at 500× g for 10 min at 4°C and the post-nuclear supernatants were spun at 27,000× g for 30 min at 4°C. The resulting crude membrane pellets were suspended in 20 mM HEPES buffer (pH 7.4) and stored at −80°C until use.

### Fatty acid extraction and analysis

Lipids were extracted from membrane pellets by chloroform∶methanol (2∶1 v/v) according to the method of Folch et al [54]. Methyl esters were prepared by 14% boron trifluoride methanolysis according to the method of Morrison and Smith [55]. Fatty acid composition was determined by gas chromatography on an Agilent 6890 Series GC (Santa Clara, CA) equipped with a flame ionization detector and a J&W Scientific DB-225 (30 m×0.25 mm I.D., 0.15 µm) capillary column (Folsom, CA). The oven temperature was ramped from an initial temperature of 100°C to 167°C at a rate of 10.65°C/min, then raised from 167°C to 206°C at a rate of 2.58°C/min and held for 8 min. Fatty acids were identified by matching retention times with known standards.

### Intracellular Ca^2+^ measurement

PHM1-41 cells were plated at a density of 8×10^4^ cells/dish in 35 mm uncoated glass-bottom dishes (MatTek, Ashland MA). After 4 days in culture, the cells were washed three times with fluorescence buffer (145 mM NaCl, 5 mM KCl, 1 mM Na_2_HPO_4_, 0.5 mM MgCl_2_, 1 mM CaCl_2_, 10 mM HEPES, 5 mM glucose, pH 7.4) and loaded with 5 µM of Fura-2-acetoxymethylester (Fura-2 AM, Invitrogen, Carlsbad, CA) for 30 min. Cells were then washed three times with fluorescence buffer and allowed to hydrolyze Fura-2 AM for 30 min. After a final set of washes in fluorescence buffer, 20–25 individual cells were analyzed for changes in intracellular free calcium [Ca^2+^]_i_ in response to 25 nM oxytocin. [Ca^2+^]_i_ was measured using an InCyt2 Im2 imaging system with excitation at 340 nm and 380 nm and emission at 510 nm (Intracellular Imaging, Cincinnati OH). Data are expressed as the average peak increase in [Ca^2+^]_i_ per dish.

### Phosphatidylinositol turnover

PHM1-41 cells were seeded at a density of 1.2×10^5^ cells/well in six-well tissue culture plates (Corning, Acton, MA). After 3 days, cells were labeled with 0.4 µM [^3^H]-myoinositol in serum-free DMEM overnight. The cells were washed twice with PBS then incubated in 1 ml of HBSS containing 10 mM LiCl and 0.1% BSA for 30 min at 37°C. Cells were treated with 100 nM oxytocin in HBSS or HBSS vehicle. Following 30 min incubation at 37°C, the media was aspirated and 1 ml of 20 mM cold formic acid was added. The cells were incubated for 30 min at 4°C then the acid was neutralized with 370 ul of 150 mM NH_4_OH. [^3^H]-inositol phosphates (IP, IP_2_, IP_3_) were isolated from cell lysates by ion exchange chromatography using AG1-X8 resin (100–200 mesh formate form, Bio-Rad, Hercules, CA) and increasing concentrations of ammonium formate in 0.1 N formic acid as described previously [56]. Data are expressed as agonist dependent activity.

### Oxytocin receptor binding assay

Total cell membrane pellets were quickly thawed at 37°C and centrifuged at 20,000× g for 30 min at 4°C. Membrane pellets were resuspended in membrane buffer (50 mM Tris, 10 mM MgCl_2_, pH 7.4) by homogenizing on ice. [tyrosyl-2,6-^3^H]-oxytocin was diluted in ligand buffer (50 mM Tris, 0.1% BSA, 10 mM MgCl_2_, pH 7.4). 96-well Multiscreen FB filter plates (Millipore, Bedford, MA) were pre-wet with 100 µl of membrane buffer for 30 min. The wetting buffer was removed by vacuum filtration and 100 µl of membrane sample was added to each well along with 100 µl of appropriate concentrations of [tyrosyl-2,6-^3^H]-oxytocin. Plates were incubated on a reciprocal shaker for 2 hr at room temperature. The binding reaction was stopped by rapid filtration under vacuum to separate bound ligand from free ligand. The plates were washed twice with 200 µl of cold wash buffer (50 mM Tris, 0.1% BSA, pH 7.4). The filters containing the bound ligand were punched out and total binding was determined by liquid scintillation counting (Scintisafe Econo 1 in Packard Tri-carb 2900TR). Nonspecific binding was determined by carrying out the binding with 10 µM of unlabeled oxytocin in the reaction mixture. The level of specific binding was calculated as the difference between total and nonspecific levels of binding. Oxytocin receptor maximal binding capacity (B_max_) and affinity (K_d_) were estimated using a one-site binding model (GraphPad Software, San Diego CA).

### Oxytocin receptor internalization assay

PHM1-41 cells were seeded into six-well tissue culture plates (Corning) at ∼1.2×10^5^ cells/well. After 4 days in culture, cells were washed twice with chilled PBS and incubated for 3 hr at 4°C in 1 mL of Ca^2+^ free HBSS containing 5 mM MgCl_2_, 0.1% BSA, and 10 nM [tyrosyl-2,6-^3^H]-oxytocin. Following 3 hr equilibration at 4°C, the cells were incubated at 37°C for specific time points, the HBSS was removed, the cells were washed twice with chilled HBSS (Ca^2+^ free, Mg^2+^ free, 0.1% BSA), then washed with 400 µL of membrane wash solution (150 mM NaCl, 50 mM acetic acid, pH 2.8) to collect membrane bound receptors. To collect internalized receptors, cells were next lysed with 400 µL of 1 M HCl and neutralized with 400 µL of 1 M NaOH. Total binding was determined by liquid scintillation counting (Scintisafe Econo 1 in Packard Tri-carb 2900TR). Nonspecific binding wells contained an additional 1 µM of unlabeled oxytocin. The level of specific binding was calculated as the difference between total and nonspecific levels of binding. Internalized receptors at each incubation time were expressed as a percentage of total receptors (specific counts in cell lysate/total of specific membrane-bound and cell lysate counts).

### Data statistical analysis

Data are expressed as mean ± SEM. Data were tested for normality using the Shapiro-Wilk test and analyzed using a linear mixed model procedure with day of experiment as a random effect (SAS/STAT 9.1, SAS Institute, Cary NC). Significance was defined as p≤0.05.

## Results

### Fatty acid incorporation into membrane

**Figure 1:**
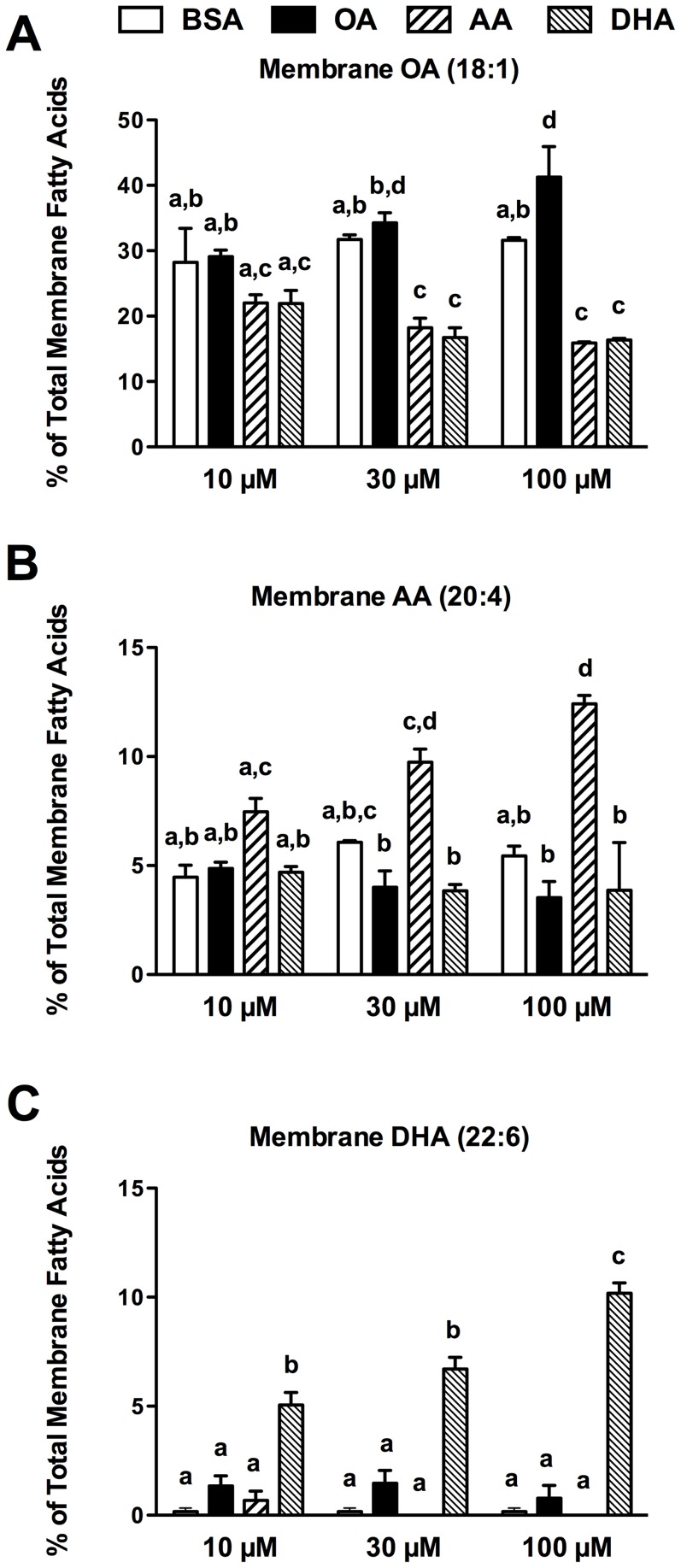
Membrane fatty acid composition of PHM1-41 cells treated with 10 µM, 30 µM, 100 µM of OA, AA, DHA. Membrane (A) 18∶1 OA, (B) 20∶4 AA, (C) 22∶6 DHA are shown as a percent of total membrane fatty acids. Values are mean percent ± SEM (n = 3 experiments). Bars not sharing common letters are significantly different between treatment groups, p<0.05.

Initial experiments determined that there was no difference in membrane FA composition of cells grown in DMEM containing 10% serum with or without the addition of FA-free BSA. The addition of exogenous fatty acids to the growth media resulted in incorporation and enrichment of that particular fatty acid in the membrane lipid of PHM1-41 cells (Figure 1). Supplementing cells with 10 µM AA resulted in a small, but nonsignificant, increase in membrane AA as a proportion of total fatty acids as compared to equimolar BSA supplementation. With 10 µM DHA supplementation there was a marked increase in membrane DHA because endogenous DHA is very low in these cells under normal culture conditions. Increasing the concentration of exogenous AA or DHA to 30 µM and 100 µM significantly enriched the respective fatty acid in the membrane as compared to equimolar BSA supplementation. OA is among the most common membrane fatty acids in PHM1-41 cells (and the FBS in the growth media); therefore significant enrichment with OA supplementation was not observed at concentrations below 100 µM. The incorporation of exogenous fatty acids appeared to be dose dependent, as membrane lipids were more enriched at 100 µM supplementation than at 10 µM supplementation.

An increase in the relative proportion of one fatty acid was accompanied by a decrease in the relative proportion of one or more other fatty acids. The relative increases in membrane AA or DHA in PUFA supplemented cells came at the expense of endogenous membrane OA. Increases in membrane 16∶0 palmitic acid and 18∶0 stearic acid also contributed to the decrease in membrane OA in PUFA supplemented cells (data not shown). OA accounted for 30±2.7% of total membrane fatty acids in cells grown in DMEM +10% FBS. In comparison, OA accounted for 18.2±1.6% and 16.7±1.6% of total membrane fatty acids in 30 µM AA and DHA treated cells and 15.9±0.2% and 16.4±0.3% in 100 µM AA and DHA treated cells, respectively. There is no significant difference in membrane OA between 30 µM and 100 µM PUFA treated cells, suggesting that the differences in oxytocin signaling observed between these two concentrations are not due to depletion of OA, but rather due to enrichment of AA or DHA in the membrane.

### Myometrial cell intracellular Ca^2+^

**Figure 2:**
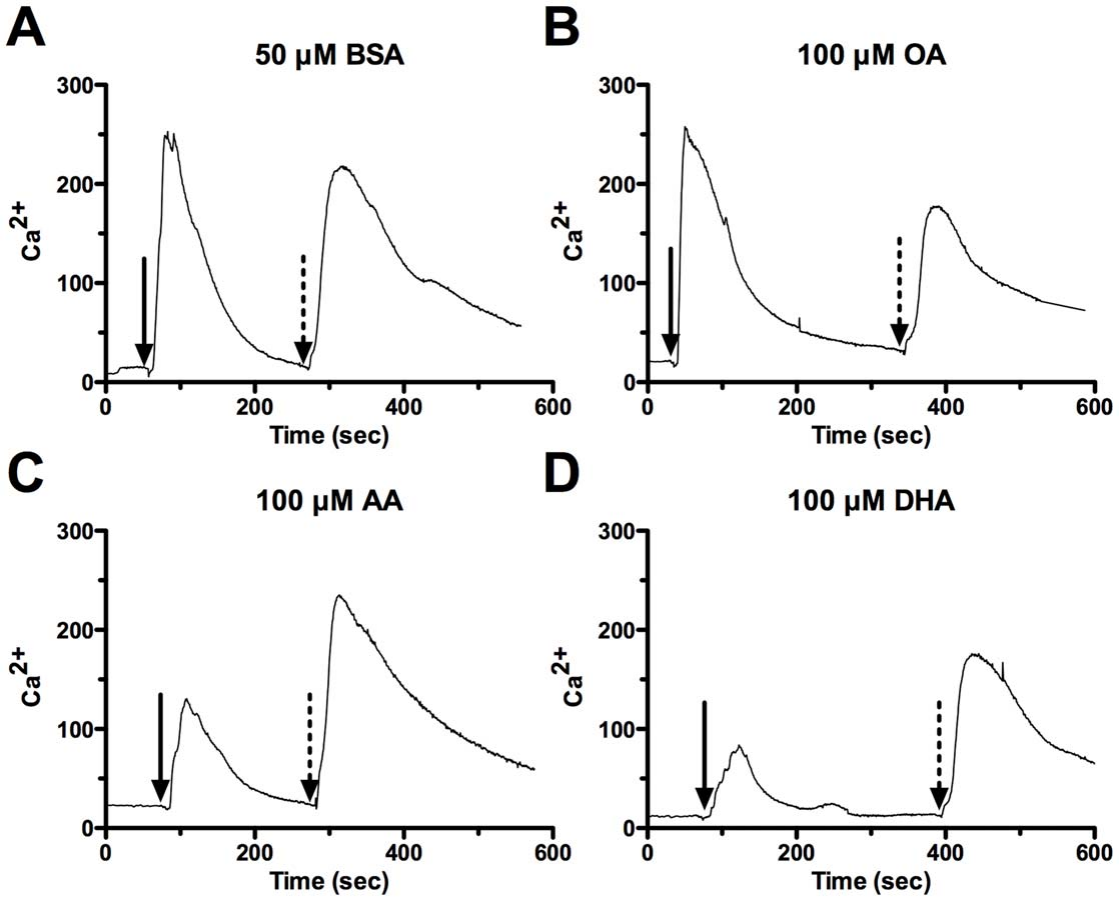
Representative [Ca^2+^]_i_ traces recorded from PHM1-41 cells. Cells were treated with (A) 50 µM BSA, (B) 100 µM OA, (C) 100 µM AA, (D) 100 µM DHA. Arrows indicate application of 25 nM oxytocin (solid) and 100 nM thapsigargin (dashed). Values are mean of 23–26 cells.

**Figure 3:**
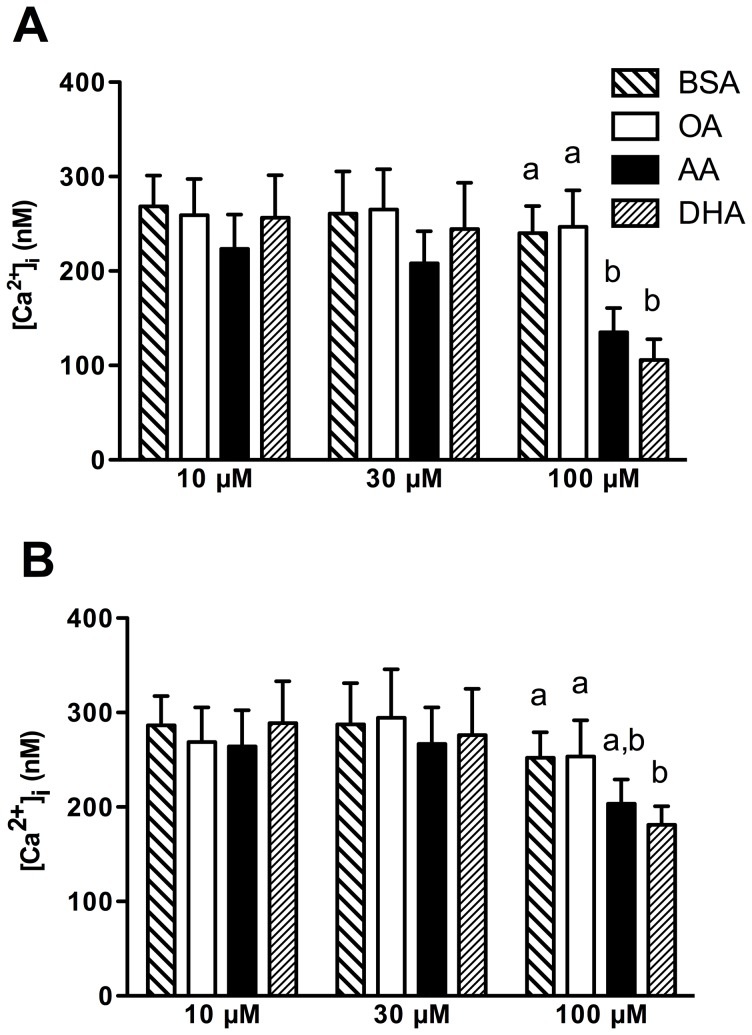
The change in [Ca^2+^]_i_ of PHM1-41 cells in response to 25 nM oxytocin. The response of all cells are shown in (A) and cells unresponsive to oxytocin stimulation are excluded from the analysis in (B). Data are expressed as the mean peak increase in [Ca^2+^]_i_ per dish. Values are mean ± SEM (n = 7 experiments at 10 µM and 30 µM; n = 12 experiments at 100 µM). Bars not sharing common letters are significantly different between treatment groups, p<0.05.

Oxytocin has been previously shown to rapidly increase [Ca^2+^]_i_ in PHM1-41 and primary myometrial cells [57]. We assessed the effect of the changes in membrane composition of PHM1-41 cells by measuring transient increases in [Ca^2+^]_i_ in response to 25 nM oxytocin and 100 nM thapsigargin stimulation (Figure 2). Thapsigargin releases [Ca^2+^]_i_ by inhibiting sarco/endoplasmic reticulum intracellular Ca^2+^-ATPase pumps. No significant differences were found in thapsigargin-induced [Ca^2+^]_i_ release between treatment groups. There were no significant differences in oxytocin-induced [Ca^2+^]_i_ mobilization between BSA and OA treatments. In contrast, oxytocin-induced [Ca^2+^]_i_ mobilization was significantly decreased 39% and 42% with 100 µM AA and 57% and 60% with 100 µM DHA treatments compared to equimolar BSA or OA treatments, respectively (Figure 3A).

Almost all cells cultured in BSA that were selected for imaging responded to oxytocin with an increase in [Ca^2+^]_i_. However, we observed considerable heterogeneity in the response of PUFA treated cells, i.e., some cells in the dish responded robustly, some weakly, and some not at all. When individual cells that exhibited no response to oxytocin were excluded from the analysis, the effect of 100 µM DHA treatment was less pronounced, but still represented a significant decrease of 25% compared to equimolar BSA or OA treatments, whereas 100 µM AA treatment was not significantly different (Figure 3B). Thus, the decrease in oxytocin-induced [Ca^2+^]_i_ mobilization observed with 100 µM PUFA could be partially attributed to a decrease in the proportion of oxytocin-sensitive cells as well as to attenuated oxytocin-mediated Ca^2+^ signaling in responsive cells.

### Phosphatidylinositol turnover

**Figure 4:**
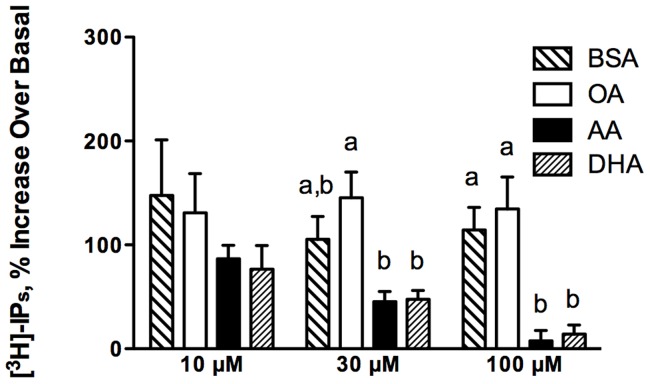
Total inositol phosphates (IPs) generated in response to 100 nM oxytocin in PHM1-41 cells. Data are expressed as a percent increase of total IPs over unstimulated basal. Values are mean ± SEM (n = 4 experiments). Bars not sharing common letters are significantly different between treatment groups, p<0.05.

Since oxytocin can mobilize [Ca^2+^]_i_ as a consequence of production of the second messenger IP_3_ [58], we evaluated the effect of membrane lipid composition on phosphatidylinositol (PI) turnover (Figure 4). Treatment with 10 µM fatty acids had no effect on total [^3^H]-inositol phosphates generated in response to 25 nM oxytocin. Treatment with 30 µM PUFA significantly decreased PI turnover compared to equimolar OA, but not BSA (AA, p = 0.12; DHA, p = 0.11) treatment. Treatment with 100 µM AA and 100 µM DHA significantly decreased PI turnover by 53% and 50% respectively as compared to equimolar BSA treatment. There were no significant differences between BSA and OA treatment at any concentration.

### Oxytocin receptor ligand binding and internalization

**Figure 5:**
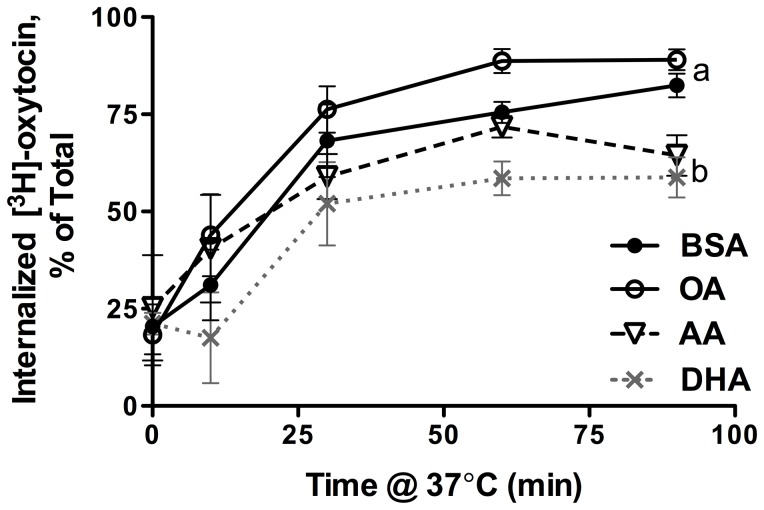
Internalized [^3^H]-oxytocin in PHM1-41 cells. Data are expressed as a percent of total cell-associated ligand (membrane bound+internalized). Values are mean ± SEM (n = 3 experiments). Points not sharing common letters are significantly different between treatment groups, p<0.05.

Treatment with 10 µM PUFA had no effect on the number of oxytocin receptor binding sites (B_max_). Treatment with 30 µM and 100 µM PUFA significantly decreased B_max_ in a dose dependent manner without affecting affinity (K_d_) compared to equimolar BSA or OA treatments (Table 1). There were no significant differences in B_max_ or K_d_ between BSA and OA treatment at any concentration. The B_max_ (3–4 pmol/mg protein) and K_d_ (≈1 nM) of controls were within the range of previously published values for the oxytocin receptor in human term myometrium [48], [59]. Treatment with PUFAs did not significantly alter the internalization of the oxytocin receptor (Figure 5). There was less ligand bound overall in 100 µM AA and 100 µM DHA treated cells (data not shown), reflecting the reduced number of oxytocin receptor binding sites, but the rate of internalization between 0 min and 30 min time points did not change compared to equimolar BSA treated cells. Maximum internalization was observed between 30 min and 60 min time points.

**Table 1:** Maximal oxytocin binding capacity (B_max_) and binding affinity (K_d_) in crude PHM1-41 cell membrane preparations.

	10 µM		30 µM		100 µM	
	B_max_	K_d_	B_max_	K_d_	B_max_	K_d_
**BSA**	473±75	0.53±0.12	437±53^a^	0.86±0.43	360±82^a^	0.43±0.19
**OA**	342±65	0.30±0.13	413±42^a^	0.48±0.18	449±103^a^	0.73±0.36
**AA**	330±71	0.38±0.12	205±66^b^	0.35±0.26	22±13^b^	0.25±0.12
**DHA**	358±90	1.22±0.53	166±61^b^	1.02±0.76	47±3^b^	0.63±0.05

B_max_ and K_d_ data are expressed as fmol/100 µg protein and nM respectively. Values are mean ± SEM (n = 3 experiments). Values not sharing common letters are significantly different between treatment groups, p<0.05.

## Discussion

To our knowledge, the present study is the first to apply LC PUFAs to the culture medium of human myometrial myocytes. Various investigators have incubated isolated cardiomyocytes [60], [61], vascular myocytes [62], and colonocytes [63] using LC n-3 PUFAs bound to albumin at concentrations ranging from 5 µM to 214 µM and exposure durations ranging from 24 h to 96 h. Our treatment regimen of 10 µM to 100 µM fatty acid incubation for 48 h falls within the range of these previous reports and we detected no impact on cell viability or morphology at these low micromolar concentrations. In the last trimester of pregnancy, maternal plasma free (non-esterified) fatty acid values of AA and DHA are 16 µM and 15 µM, respectively [64]. Most of the human plasma AA and DHA reside in esterified forms, mainly as phospholipid constituents of lipoproteins [65]. This phospholipid pool is generally overlooked in terms of fatty acid transport. Nevertheless, the uptake and hydrolysis of liproproteins such as LDL would act as an in vivo delivery mechanism for not only cholesterol but also for the fatty acids of the LDL particle, contributing to the amount of fatty acids transported in the free pool. Thus the concentrations employed in the present study are within the physiological range.

Several studies have implicated eicosanoids in the regulation of gestational duration, parturition and the initiation of labor [5], [16], [32], [33]. Both PG and leukotrienes (LT), such as PGE_2_, PGF_2α_, LTC_4_, and LTB_4_ increase prior to the onset of labor. Exogenous PGE_2_ or PGF_2α_ provision induces cervical ripening and uterine contractions in both full-term and preterm labor [22]–[25]. Selective inhibitors of cyclooxygenase-2 are effective in decreasing PGE_2_ in amniotic tissues and have been explored for preventing preterm deliveries, although there are many complicating issues. PGE_2_ levels in amnion, chorion, decidua and myometrium from women in labor, whether preterm or term, were 2- to 5- fold higher than those in samples from nonlaboring women [33]. However, PGE_2_ levels in women delivering preterm, whether in labor or not in labor, were markedly lower than either laboring or nonlaboring women delivered at term [33]. Another study of PG concentrations in labor has reported that both PGE2 and PGF2_α_ levels were significantly lower in amnion and placenta in women delivered preterm than in women delivered at term [32].

Considering these observations, it is difficult to reconcile the inhibitory effect of LC n-3 PUFAs on PG biosynthesis [28], [29] as the sole mechanism of action for DHA in prolonging gestation. Besides their effects on production of PGs, LC n-3 PUFAs can act directly at the plasma membrane level. This was first demonstrated by Leaf et al regarding the antiarrhythmic effect of fish and fish oil consumption [34]. The precise site of action in cardiomyocytes remains to be fully elucidated, but data from Anderson and colleagues [37] suggest that LC n-3 PUFAs may electrically stabilize cardiomyocytes by modulating ion channels through changes in membrane fluidity and elasticity in the microdomain bordering the transmembrane protein.

LC n-3 PUFAs have also been shown to exert their anti-inflammatory properties through eicosanoid-independent membrane effects. The successful application of LC n-3 PUFAs in the treatment of immune-inflammatory diseases is based largely on their ability to suppress T lymphocyte activation [66], [67]. A number of key proteins involved in signal transduction are known to associate with lipid rafts, targeted there by acylation with saturated fatty acids [68]. LC n-3 PUFAs have been proposed to disrupt T cell activation by displacing raft-resident signaling proteins such as Src family kinases and transmembrane linker for activation of T cells from lipid rafts [38], [39]. LC n-3 PUFAs are thought to act primarily by altering raft composition and structure, although changes in protein fatty acylation may contribute to the displacement of proteins from lipid rafts [69]. These observations raise the possibility that dietary LC n-3 PUFAs prolong gestational duration through changes in the lipid bilayer properties and associated changes in signaling mechanisms.

In the present study we demonstrated that both AA and DHA significantly alter the membrane lipid composition of PHM1-41 pregnant human myometrial smooth muscle cells (Figure 1). We are not aware of any fish oil supplementation trials in pregnant women that have characterized myometrial membrane fatty acid composition. Data for maternal erythrocyte phospholipids does exist for an approximate comparison. Dunstan et al have reported increases in maternal erythrocyte DHA as a percentage of total fatty acids at 37 weeks gestation, from 6.6±0.2% in the control group to 11.0±0.2% with fish oil consumption equivalent to one fatty fish meal per day (total of 2.2 g DHA and 1.1 g EPA per day) [70]. In the present study, PHM1-41 membrane DHA composed 6.7±0.5% of total fatty acids with 30 µM DHA treatment and 10.2±0.5% with 100 µM DHA treatment. Relative proportions of other fatty acids were similar between erythrocytes from women fed fish oil and DHA treated PHM1-41 cells. Stearic acid and OA each composed 14–16% of total fatty acids in both erythrocytes and in myocytes treated with 30 µM and 100 µM DHA.

The increase in membrane AA and DHA was associated with suppressed oxytocin-stimulated [Ca^2+^]_i_ mobilization without affecting the size of the releasable thapsigargin-sensitive [Ca^2+^]_i_ store (Figures 2 and 3). Since the cells were washed several times during the Ca^2+^ dye loading procedure, an interpretation based on a direct effect of free fatty acid as proposed in cardiomyocytes and rat aortic smooth muscle cells [71] appears unlikely. Inhibition of the oxytocin-stimulated rise in [Ca^2+^]_i_ was associated with near abolition of PI turnover (7.9±9.7% and 14.2±8.8% over basal for 100 µM AA and 100 µM DHA treated cells, respectively) (Figure 4).

Upstream of PI turnover, we found that 100 µM LC PUFA reduced the number of oxytocin receptor binding sites in the membrane without affecting binding affinity (Table 1). Fahrenholz et al have demonstrated that membrane cholesterol acts as an allosteric modulator of the oxytocin receptor [40], [41]. High-affinity state receptors are stabilized three-fold (t_1/2_) and enriched two-fold in cholesterol-rich, caveolin-containing membrane microdomains [42]. Furthermore, oxytocin receptors are known to initiate different signaling pathways, stimulating or inhibiting cell growth, depending on their location inside or outside caveolar microdomains (43,44). The range of oxytocin concentrations in our saturation binding study (10^−10^ to 10^−7^ M) would not detect a second, low affinity binding site in the 10^−7^ to 10^−6^ M range as reported by Zhong et al [72]. Since Chen et al have shown that DHA incorporation into membrane phospholipids depletes cholesterol from caveolae [73], the possibility that DHA in the present study shifts a population of receptors into the low affinity binding state may warrant further investigation. PI turnover and receptor binding sites decreased with 30 µM PUFA treatment, but remained at levels sufficient for oxytocin-stimulated [Ca^2+^]_i_ mobilization under these conditions.

Prolonged exposure to agonists desensitizes many G protein-coupled receptors (GPCRs), protecting the cell from overstimulation. Following receptor activation, GPCRs are commonly subject to a cycle of internalization and sequestration, resensitization, and recycling back to the plasma membrane [74], [75]. It is unlikely that the alteration of membrane fatty acid composition affected receptor internalization, however, as we did not detect any differences in the rate of oxytocin receptor internalization upon ligand binding (Figure 5).

In view of the frequently antagonistic actions of dietary AA and DHA, our observation that both n-6 and n-3 LC PUFAs inhibit oxytocin signaling was unexpected. Although we have discovered a generalized LC PUFA effect, translation to human diets and commonly consumed supplements would indicate that this mechanism of action would pertain to fish consumption and fish oil supplementation. AA in the body originates from either synthesis through desaturation and elongation of 18∶2 linoleic acid (LA), an essential fatty acid, or as a result of dietary intake. The intake of LA contributes 5–7% of dietary energy in western nations. A recent review of human literature found that increasing the contribution of LA to as much as 20% of dietary energy does not increase tissue AA in erythrocytes and plasma/serum phospholipids [76]. Meat, fish, and eggs are the major food sources of preformed AA. Studies comparing the fatty acid profile of omnivores and vegetarians have reported only slight differences in the level of AA [77], [78].

These data suggest that a moderate variation in intake of AA has no major effect on AA accumulation; thus an effect of AA supplementation observed in cell culture may not be relevant to a physiological diet. In contrast, it is well established that human phospholipid LC n-3 PUFA, such as DHA, responds to dietary intake of DHA and that high linolenic acid intake does not increase phospholipid DHA [79]. It is also important to note that DHA (and also EPA) supplementation displaces AA from human plasma phospholipids in human volunteers consuming a normal Western diet (77).

In conclusion, we have shown that LC PUFAs inhibit oxytocin-stimulated [Ca^2+^]_i_ mobilization in a cell culture model. These findings, in conjunction with the demonstrated ability of dietary LC PUFAs to alter lipid composition, provide new insight into possible mechanisms pertaining to reports of dietary fish and fish oil consumption prolonging gestation.
